# Structure of a catalytic dimer of the α- and β-subunits of the F-ATPase from *Paracoccus denitrificans* at 2.3 Å resolution

**DOI:** 10.1107/S2053230X15016076

**Published:** 2015-09-23

**Authors:** Edgar Morales-Ríos, Martin G. Montgomery, Andrew G. W. Leslie, José J. García-Trejo, John E. Walker

**Affiliations:** a The Medical Research Council Mitochondrial Biology Unit, Cambridge Biomedical Campus, Hills Road, Cambridge CB2 0XY, England; b The Medical Research Council Laboratory of Molecular Biology, Cambridge Biomedical Campus, Francis Crick Avenue, Cambridge CB2 0QH, England; c Departmento de Biología, Facultad Química, Universidad Nacional Autónoma de México, Mexico City, Mexico

**Keywords:** α-proteobacteria, *Paracoccus denitrificans*, F-ATPase, structure, catalytic αβ dimer

## Abstract

The structures of F-ATPases have predominantly been determined from mitochondrial enzymes, and those of the enzymes in eubacteria have been less studied. *Paracoccus denitrificans* is a member of the α-proteobacteria and is related to the extinct protomitochondrion that became engulfed by the ancestor of eukaryotic cells. The *P. denitrificans* F-ATPase is an example of a eubacterial F-ATPase that can carry out ATP synthesis only, whereas many others can catalyse both the synthesis and the hydrolysis of ATP. Inhibition of the ATP hydrolytic activity of the *P. denitrificans* F-ATPase involves the ζ inhibitor protein, an α-helical protein that binds to the catalytic F_1_ domain of the enzyme. This domain is a complex of three α-subunits and three β-subunits, and one copy of each of the γ-, δ- and ∊-subunits. Attempts to crystallize the F_1_–ζ inhibitor complex yielded crystals of a subcomplex of the catalytic domain containing the α- and β-subunits only. Its structure was determined to 2.3 Å resolution and consists of a heterodimer of one α-subunit and one β-subunit. It has no bound nucleotides, and it corresponds to the ‘open’ or ‘empty’ catalytic interface found in other F-ATPases. The main significance of this structure is that it aids in the determination of the structure of the intact membrane-bound F-ATPase, which has been crystallized.

## Abstract 2

The structure of the αβ heterodimer of the F-ATPase from the α-proteobacterium *P. denitrificans* has been determined at 2.3 Å resolution. It corresponds to the ‘open’ or ‘empty’ catalytic interface found in other F-ATPases.

## 1. Introduction   

The structures and mechanisms of F-ATPases from eubacteria, chloroplasts and mitochondria have many common features in their structures and mechanisms. Our current knowledge of how they function by a rotary mechanism is based largely on the knowledge of the structures of mostly mitochondrial enzymes (Walker, 2013 ▸; Robinson *et al.*, 2013 ▸; Bason *et al.*, 2014 ▸, 2015 ▸) and ‘single-molecule’ experiments conducted almost entirely on enzymes from *Escherichia coli* and *Bacillus stearothermophilus* (or *Geobacillus stearo­thermophilus*) strain PS3 (Watanabe & Noji, 2013 ▸). For example, more than 25 high-resolution structures of the F_1_ catalytic domain from bovine mitochondria with bound substrates, substrate analogues and inhibitors have been described (Walker, 2013 ▸; Robinson *et al.*, 2013 ▸; Bason *et al.*, 2014 ▸, 2015 ▸). In contrast, there are two structures of the F_1_ catalytic domain of the *E. coli* enzyme (Cingolani & Duncan, 2011 ▸; Roy *et al.*, 2012 ▸) and one of the same domain of the enzyme from *B. stearothermophilus* (Shirakihara *et al.*, 2015 ▸), and another of the α_3_β_3_ subcomplex derived from the F_1_ domain (Shirakihara *et al.*, 1997 ▸), plus a structure of F_1_-ATPase from *Caldalkalibacillus thermarum* (Stocker *et al.*, 2007 ▸). In addition, the structures of c-rings from the rotors of several eubacterial species have been determined at high resolution in isolation from the rest of the complex (Meier *et al.*, 2005 ▸; Pogoryelov *et al.*, 2009 ▸; Preiss *et al.*, 2013 ▸, 2014 ▸; Matthies *et al.*, 2014 ▸). There is also fragmentary structural information concerning the peripheral stalk region of the F-ATPase from *E. coli* determined by nuclear magnetic resonance in solution, the N-terminal domain of the δ-subunit and its mode of interaction with the N-terminal region of an α-subunit (Wilkens *et al.*, 2005 ▸), and for segments of the β-subunit (Dmitriev *et al.*, 1999 ▸; Del Rizzo *et al.*, 2002 ▸; Priya *et al.*, 2009 ▸). Part of the reason for this relative dearth of structural information on the catalytic domain of bacterial F-ATPases is that the F_1_ domain of the enzyme from *E. coli*, for example, is rather unstable under the conditions that have been employed for crystallizing mitochondrial enzymes. Also, there is no generic method for purifying eubacterial F-ATPases, whereas it has been demonstrated that mitochondrial enzymes can be purified from a wide range of species by affinity chromatography with the inhibitory region of bovine IF_1_, the protein inhibitor of the bovine mitochondrial F-ATPase (Runswick *et al.*, 2013 ▸; Walpole *et al.*, 2015 ▸; Liu *et al.*, 2015 ▸). Therefore, we have decided to explore the possibility of developing the F-ATPase from *Paracoccus denitrificans* as a subject for structural analysis. *P. denitrificans* is a member of the bacterial class α-proteobacteria in the phylum Proteobacteria. The class includes the extinct protomitochondrion that became engulfed by the ancestor of eukaryotic cells, and the respiratory chain of *P. denitrificans* has been recognized as being especially similar to respiratory chains in mitochondria (John & Whatley, 1975 ▸).

Some eubacterial F-ATPases, exemplified by those from *E. coli* and *B. stearothermophilus*, can synthesize ATP from ADP and phosphate using the transmembrane proton motive force as a source of energy, and under anaerobic conditions can operate in reverse and use the energy released by the hydrolysis of ATP made by glycolysis to generate a transmembrane proton motive force. Other eubacterial F-ATPases, exemplified by those from *C. thermarum* (Cook *et al.*, 2003 ▸) and *P. denitrificans* (Zharova & Vinogradov, 2012 ▸), can synthesize ATP in the presence of a proton motive force, but their ATP hydrolase activity is inhibited in its absence (Pacheco-Moisés *et al.*, 2000 ▸, 2002 ▸). The mechanism of inhibition in *C. thermarum* is not understood, but in *P. denitrificans* and other α-proteobacteria the inhibition of ATP hydrolysis involves an inhibitor protein known as the ζ inhibitor protein (Morales-Ríos *et al.*, 2010 ▸). This inhibitor protein has not been detected in other classes of bacteria. The structure of the free ζ inhibitor is known from studies employing nuclear magnetic resonance in solution (Serrano *et al.*, 2014 ▸). It binds to the F_1_ catalytic domain of the F-ATPase and can be cross-linked covalently to the α-, β-, γ- and ∊-subunits (Zarco-Zavala *et al.*, 2014 ▸). However, the cross-linked residues were not identified, and its mode of interaction with this domain is not known.

Therefore, we have purified the F_1_-ATPase from *P. denitrificans* with the ζ inhibitor protein bound to it, and a second complex devoid of the ∊-subunit, known as F_1_–ζ and F_1_Δ∊–ζ, respectively. As in other species where the subunit composition of the F_1_ domain has been established experimentally, the F_1_ domain in *P. denitrificans* is probably an assembly of three α-subunits and three β-subunits, where the catalytic sites are found, plus one copy of each of the γ-, δ- and ∊-subunits, with the γ- and ∊-subunits forming the central rotor of the enzyme penetrating along the central axis of the α_3_β_3_ domain, and the δ-subunit, a residual component of the peripheral stalk in the intact F-ATPase, sitting ‘on top’ of the α_3_β_3_ domain. In the bovine F_1_-ATPase, for example, the three catalytic sites are found at three of the six interfaces between α- and β-subunits, known as the ‘catalytic interfaces’. The asymmetry of the central stalk imposes different conformations on the three catalytic sites. In a ground-state structure of the catalytic domain (Abrahams *et al.*, 1994 ▸; Bowler *et al.*, 2007 ▸), two of them, the β_DP_ and the β_TP_ sites, have similar, but significantly different, closed conformations. Both bind nucleotides, but catalysis occurs at the β_DP_ site. The third, or β_E_, site has a different open conformation with low nucleotide affinity. These three catalytic conformations correspond to ‘tight’, ‘loose’ and ‘open’ states in a binding-change mechanism of ATP hydrolysis and synthesis (Boyer, 1993 ▸).

As described here, we have attempted to crystallize the F_1_–ζ and F_1_Δ∊–ζ complexes. Crystals were obtained for the F_1_Δ∊–ζ complex, but none were obtained for the F_1_–ζ complex. However, as described below, the crystals with F_1_Δ∊–ζ as the starting material were found to contain a heterodimer of one α-subunit and one β-subunit, which had formed by dissociation of the complex under the conditions of crystallization. This heterodimer has no bound nucleotide, and it represents the ‘open’ or ‘empty’ β_E_ catalytic interface of the intact F-ATPase.

## 2. Materials and methods   

### 2.1. Protein methods   

The protein compositions of various samples were analysed by SDS–PAGE in 12–22% polyacrylamide gradient gels (Laemmli, 1970 ▸). Proteins were stained with 0.2% Coomassie Blue dye or with silver. Protein concentrations were measured by the bicinchoninic acid method (Life Technologies, Paisley, Scotland). The latent ATP hydrolase activities of the F_1_-ATPase and of the enzyme lacking the ∊-subunit (F_1_Δ∊) from *P. denitrificans* were activated with 0.1% lauryldimethylamine oxide (LDAO) and 4 m*M* sodium sulfite, and their activities were measured by coupling them to the oxidation of NADH monitored using the absorbance of ultraviolet light at 340 nm (Pullman *et al.*, 1960 ▸).

### 2.2. Cell growth   

A starter culture of *P. denitrificans* (strain PD1222, Rif^r^, Spe^r^, enhanced conjugation frequencies, m^+^, or host-specific modification) was grown at 30°C for 18 h in 1 l Luria–Bertani medium (Miller, 1987 ▸) containing 100 µg ml^−1^ spectinomycin. It was inoculated into 70 l succinate medium consisting of 1%(*w*/*v*) sodium succinate, 50 m*M* disodium hydrogen phosphate, 1.25 m*M* magnesium chloride, 1 m*M* citric acid, 100 µ*M* calcium chloride, 90 µ*M* ferric chloride, 50 µ*M* manganese chloride, 25 µ*M* zinc chloride, 10 µ*M* cobalt chloride and 10 µ*M* boric acid. The culture was grown at 30°C for 16 h in an Applikon ADI 1075 fermenter (100 l maximum capacity). The yield of wet cells was 2 kg. Inside-out vesicles were prepared by osmotic shock (Pacheco-Moisés *et al.*, 2000 ▸).

### 2.3. Purification of the complex of the F_1_-ATPase and the ζ inhibitor protein from *P. denitrificans*   

Using modification of an earlier method (Morales-Ríos *et al.*, 2010 ▸), the F_1_–ζ inhibitor complex was released from a suspension of membranes from *P. denitrificans* (30 ml) by the addition of chloroform (15 ml). The two phases were mixed for 30 s and then centrifuged (2939*g*, 25°C). The upper aqueous phase was centrifuged again (50 min, 224 468*g*, 4°C), and the supernatant was applied to a HiTrap Q HP column (5 ml; GE Healthcare) equilibrated with purification buffer consisting of 50 m*M* Tris–HCl pH 7.5, 10%(*v*/*v*) glycerol, 0.5 m*M* ATP, 2 m*M* MgCl_2_ and protease-inhibitor tablets (cOmplete, EDTA-free; Roche; one tablet per 100 ml). The column was eluted with buffer containing a gradient of sodium chloride with steps of 50, 100, 150, 200, 225, 250, 275, 300 and 325 m*M*. The fractions (15 ml) were analysed by SDS–PAGE, and those containing the purest enzyme–inhibitor complex were pooled and concentrated (final volume 500 µl; protein concentration 15 mg ml^−1^) with a Vivaspin ultrafiltration concentrator (molecular-weight cutoff 50 kDa; 2939*g*, 15°C). The two separate concentrates of the F_1_–ζ and the F_1_Δ∊–ζ complexes (see below) were applied individually to a column of Superdex 200 (10 × 300 mm; GE Healthcare) equilibrated with purification buffer and eluted at a flow rate of 0.5 ml min^−1^. The peak fractions (3 ml) were pooled and concentrated as above (final volume 150 µl; protein concentration 10 ml min^−1^).

### 2.4. Crystallization of the catalytic dimer of α- and β-subunits of the F-ATPase from *P. denitrificans*   

The crystals were grown at 25°C by the microbatch method under oil in 72-well Nunc plates. Drops (2 µl) were formed by mixing the solution of purified F_1_Δ∊–ζ (protein concentration 10 ml min^−1^) with an equal volume of buffer consisting of 50 m*M* Tris–HCl pH 7.8, 12%(*w*/*v*) polyethylene glycol 10 000, 1%(*w*/*v*) cadaverine, 10%(*v*/*v*) glycerol, 1 m*M* ATP. They were harvested with micro-mounts (MiTeGen) and vitrified in liquid nitrogen in the presence of cryoprotection buffer consisting of 25 m*M* Tris–HCl pH 7.8, 15%(*v*/*v*) glycerol, 10%(*w*/*v*) polyethylene glycol 10 000, 1%(*w*/*v*) cadaverine. 25 crystals were washed three times in buffer with the same composition as the mother liquor and analyzed by SDS–PAGE. Similar, but unsuccessful, attempts were made to grow crystals of F_1_–ζ.

### 2.5. Data collection, structure solution and refinement   

X-ray diffraction data were collected from the cooled cryoprotected crystals using a Pilatus 6M-F detector (Dectris) on beamline I03 (wavelength 0.9763 Å; beam size 90 × 35 µm) at the Diamond Light Source, Harwell, Oxfordshire, England. The data were processed with programs from the *CCP*4 suite (Winn *et al.*, 2011 ▸). Diffraction images were integrated with *iMosflm* (Battye *et al.*, 2011 ▸) and the data were reduced with *AIMLESS* (Evans & Murshudov, 2013 ▸). Molecular replacement was carried out with *Phaser* (McCoy *et al.*, 2007 ▸) with the α_E_- and β_E_-subunits of the currently most accurate structure of bovine F_1_-ATPase (Bowler *et al.*, 2007 ▸; PDB entry 2jdi) as a template. The model was built with *Coot* (Emsley *et al.*, 2010 ▸) and refined with *REFMAC*5 (Murshudov *et al.*, 2011 ▸). The stereochemistry of the model following each round of refinement was assessed with *Coot* and *MolProbity* (Chen *et al.*, 2010 ▸). Figures were made with *PyMOL* (Schrödinger).

## 3. Results and discussion   

### 3.1. Characterization of the complex of the F_1_-ATPase and the ζ inhibitor protein from *P. denitrificans*   

**Figure 1:**
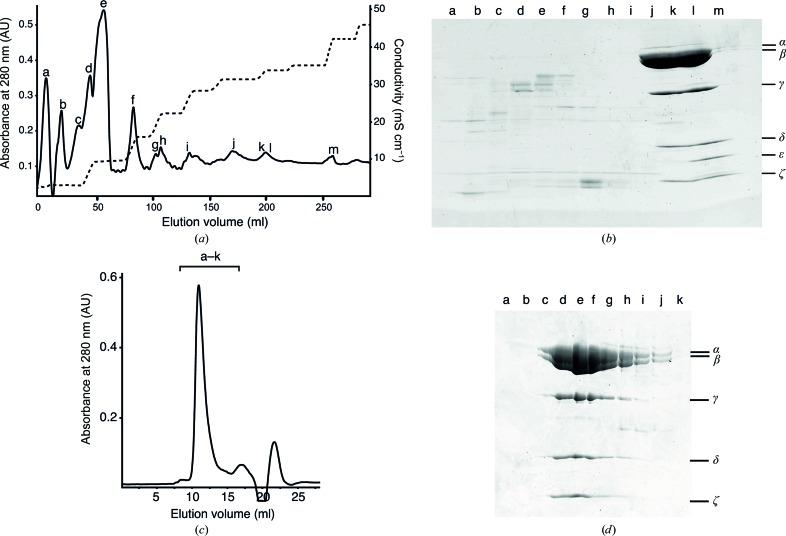
Purification of complexes of F_1_-ATPase and the ζ inhibitor protein from *P. denitrificans*. (*a*) Elution profile from a HiTrap Q column. Fractions of 5 ml were collected. The absorbance of the eluate was monitored at 280 nm (solid line) and the resistivity of the eluent was measured (dashed line). (*b*) Analysis of the protein compositions of peaks a–m in (*a*). The positions of subunits of the F_1_-ATPase and of the ζ inhibitor protein are indicated on the right. (*c*) Gel-filtration chromatography of the F_1_Δ∊–ζ complex from *P. denitrificans* [fractions j and k in (*b*)]. The absorbance of the eluate was monitored at 280 nm. The volume of each of fractions a–k (the bracketed region) was 0.5 ml. (*d*) Analysis by SDS–PAGE of fractions a–k in (*c*). The positions of subunits of the F_1_Δ∊–ζ complex are indicated on the right.

Three peaks (j, k and l in Fig. 1 ▸ *a*) containing subunits of the *P. denitrificans* F_1_-ATPase complex were eluted from the Q Sepharose column. Analysis by SDS–PAGE revealed that peak j contained a complex of the α-, β-, γ- and δ-subunits from the F_1_ domain of the F-ATPase plus the ζ inhibitor protein (the F_1_Δ∊–ζ complex), and the two subsequent peaks k and l contained a complex of the intact F_1_-ATPase with the ζ protein (the F_1_–ζ complex). The ATP hydrolase activities of the F_1_–ζ and F_1_Δ∊–ζ complexes were 0.01 ± 0.002 and 0.02 ± 0.001 U per milligram of protein, respectively, and after relief of the inhibitory activity of the inhibitor protein they were 3.5 ± 0.1 and 4 ± 0.1 U per milligram of protein, respectively. These values are comparable with those of other inhibited bacterial F-ATPases where no inhibitor protein is involved. For example, the values for the F_1_-ATPase from the cyanobacterium *Thermosynechococcus elongatus* are 0.2 and 9.0 U per milligram of protein before and after activation with LDAO (Sunamura *et al.*, 2012 ▸). For *C. thermarum* they are 0.9 and 28.5 U per milligram of protein before and after activation (Keis *et al.*, 2006 ▸), and for the chloroplast F_1_-ATPase from *Spinacia oleracea* they are 4.4 and 39.7 U per milligram of protein before and after activation (Groth & Schirwitz, 1999 ▸). Enzymes that are not inhibited in ATP hydrolysis have higher recorded values than those of the activated inhibited enzymes. Values in the range 60–130 U per milligram of protein have been reported for the F_1_-ATPase from *E. coli* (Dunn *et al.*, 1990 ▸). With the bovine F_1_-ATPase, activities in excess of 120 U per milligram of protein have been recorded routinely (van Raaij *et al.*, 1996 ▸).

The concentrated F_1_Δ∊–ζ complex was subjected to gel-filtration chromatography (Fig. 1 ▸). This experiment removed minor contaminants, and confirmed that the α-, β-, γ- and δ-subunits from the F_1_ domain of the F-ATPase, plus the ζ inhibitor protein, form an integral F_1_Δ∊–ζ complex that is stable under the conditions of chromatography (Figs. 1 ▸ *c* and 1 ▸ *d*). Other experiments (not shown) were conducted with the F_1_–ζ complex, with similar conclusions.

### 3.2. Crystallization of the dimer of the α- and β-subunits of the F-ATPase from *P. denitrificans*   

**Figure 2:**
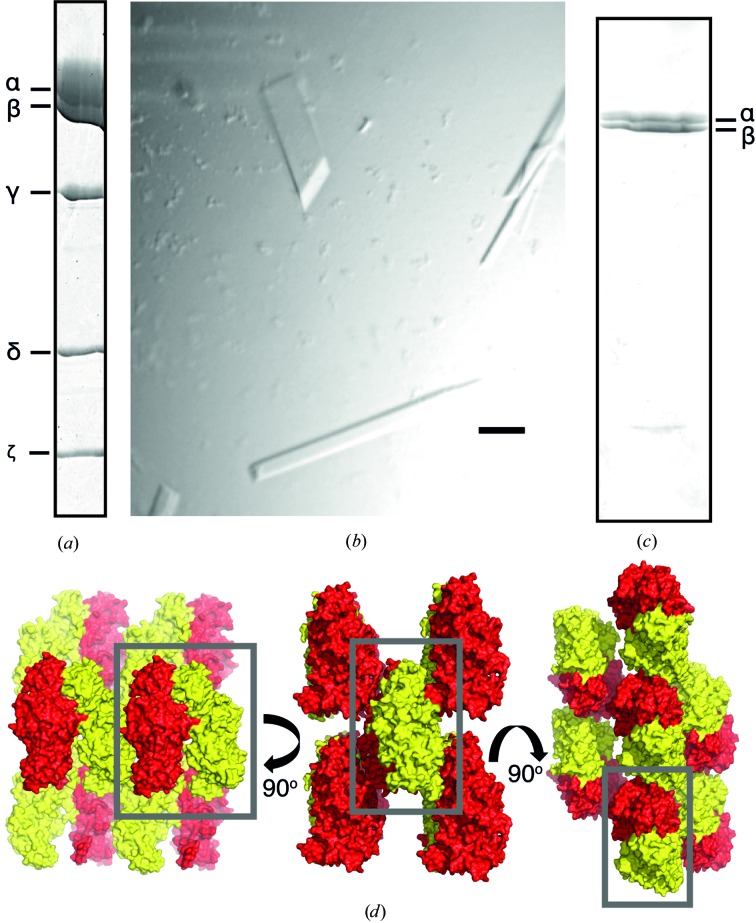
Crystals of the catalytic dimer of α- and β-subunits of the F-ATPase from *P. denitrificans*. (*a*) SDS–PAGE analysis of the F_1_Δ∊–ζ inhibited complex (15 µg) used in the crystallization experiment. (*b*) Crystals after 25 d of growth. The bar represents 100 µm. (*c*) SDS–PAGE analysis of the washed rhombic crystals [top left in (*b*)]. The positions of the α- and β-subunits of the enzyme are indicated on the right. (*d*) Packing of αβ dimers in the crystal lattice. The grey box contains an αβ dimer viewed from three aspects related by rotations of 90°.

Attempts were made to crystallize both the F_1_–ζ and F_1_Δ∊–ζ inhibited complexes. No crystals were obtained for the former, but the latter yielded crystals with two different morphologies: needles and rhomboids (Fig. 2 ▸). The rhombic crystals reached their maximum size (approximately 200 × 40 × 5 µm) after 25 d of growth at 25°C and only these crystals gave useful X-ray diffraction data. The dimensions of the unit cell calculated from the X-ray diffraction data were *a* = 72.6, *b* = 102.9, *c* = 89.2 Å, and the space group was determined as *P*2_1_. The asymmetric unit of this cell is too small to accommodate an F_1_-ATPase complex. Therefore, it seemed likely that a subcomplex of the enzyme had formed under the conditions of crystallization and the subcomplex had crystallized. This conclusion was confirmed by analysis of the rhombic crystals by SDS–PAGE, which showed that the crystals contained only α- and β-subunits (Fig. 2 ▸ *c*); presumably this subcomplex had formed by loss of the γ- and δ-subunits and dissociation of the α_3_β_3_ subcomplex during the crystallization process. At this stage, the precise composition of the subcomplex was unclear, as it could conceivably have contained one, two or three copies of each of the α- and β-subunits. Again, the size of the, α_3_β_3_ subcomplex was incompatible with the unit-cell parameters and, given that the α_2_β_2_ subcomplex has never been observed from any F-ATPase, it was most likely that the crystals were formed from one of two possible αβ hetereodimers, containing either a catalytic or a noncatalytic interface of the F_1_-ATPase.

### 3.3. Structure of the dimer of the α- and β-subunits of the F-ATPase from *P. denitrificans*   

**Figure 3:**
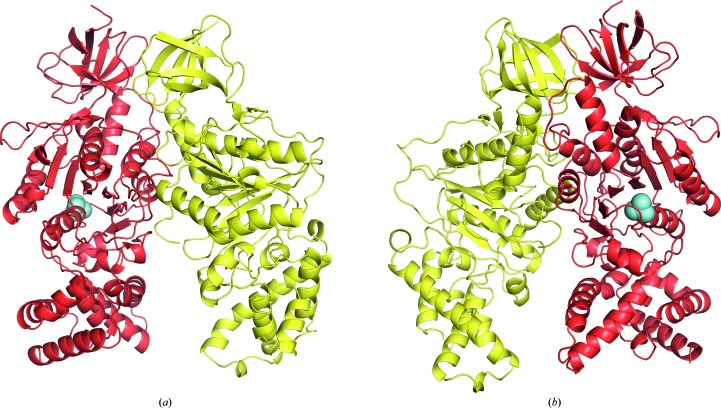
Structure of the catalytic dimer of the α- and β-subunits of the F-ATPase from *P. denitrificans*. The α- and β-subunits are shown in red and yellow, respectively, and a bound phosphate ion is denoted by cyan spheres. (*a*) View from the front, looking inwards towards the central stalk in the rotor of the intact enzyme. The arrangement of subunits, with the α-subunit on the left and the β-subunit on the right, corresponds to a catalytic interface in the intact F_1_-ATPase. (*b*) View from the inside of the intact complex, looking outwards.

The structure of the *P. denitrificans* αβ complex (Fig. 3 ▸) was solved by molecular replacement with data to 2.3 Å resolution. Both the catalytic and noncatalytic αβ dimers of bovine F_1_-ATPase were tried, but it was clear that the former was appropriate and the latter was not. The packing of the protein complexes in the crystal lattice provided additional confirmation that the crystal lattice consisted of αβ dimers and not α_3_β_3_ hexamers (Fig. 2 ▸ *d*). Data-processing and refinement statistics are summarized in Table 1 ▸. The final model contains residues 24–511 of the α-subunit and residues 4–273, 279–314 and 320–469 of the β-subunit. Associated with the structure are eight molecules of glycerol, 79 molecules of water and one phosphate ion. As in other structures of F_1_-ATPases, the α- and β-subunits of the F-ATPase from *P. denitrificans* have very similar folds (r.m.s.d. of 5.1 Å). Both are composed of three domains. The N-terminal domains (residues 24–95 in the α-subunit and residues 4–77 in the β-subunit) consist of six β-strands. In the intact enzyme in other species, alternating N-terminal domains from each of the three α- and β-subunits are hydrogen-bonded together in the stable circular ‘crown’ structure of the F_1_-ATPase. The N-terminal domains of the α- and β-subunits in the αβ dimer from *P. denitrificans* are followed by the central nucleotide-binding domains (residues 96–381 in the α-subunit and residues 78–355 in the β-subunit). They consist of ten β-strands and eight α-helices and seven β-strands and five α-helices, respectively. The remainder of the α- and β-subunits, residues 382–511 in the α-subunit and residues 356–469 in the β-subunit, are folded into a bundle of six and seven α-helices that form the C-terminal domains of the subunits.

**Figure 4:**
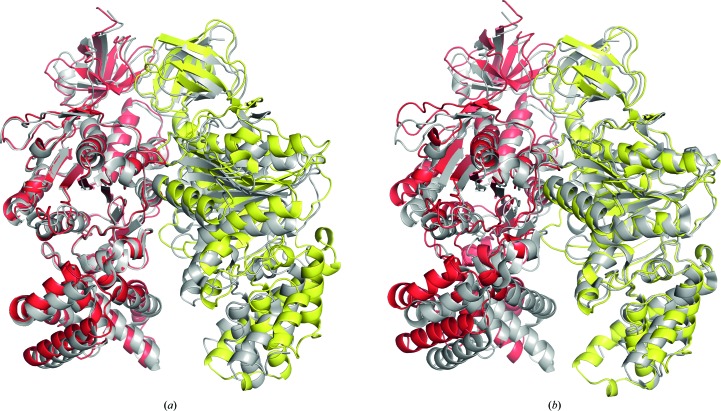
Alignment of the structures of the catalytic dimer of α- and β-subunits of the F-ATPase from *P. denitrificans* with the α- and β-subunits forming the open (or empty) catalytic interface in the ground-state structure of bovine F_1_-ATPase (grey). (*a*) and (*b*) show alignments *via* the α- and β-subunits, respectively.

Despite the presence of ATP and magnesium ions in the mother liquor during the formation of crystals, in the structure of the αβ dimer no nucleotide was found to be associated with either of the subunits. The nucleotide-binding and C-terminal domains of the β-subunit are in a conformation similar to the open or empty conformations in β_E_-subunits in almost all of the known structures of F_1_-ATPase, and therefore the αβ interface appears to correspond to the empty or open catalytic interface of the *P. denitrificans* F-ATPase. However, the α_E_β_E_ interface is more open than in bovine F_1_-ATPase because of contacts in the crystal lattice. Thus, the global r.m.s.d. of the αβ dimer from *P. denitrificans* compared with the α_E_β_E_ dimer from the bovine ground-state F_1_-ATPase is 3.0 Å (Fig. 4 ▸). The values for the α- and β-subunits alone are 2.1 and 2.0 Å, respectively.

**Figure 5:**
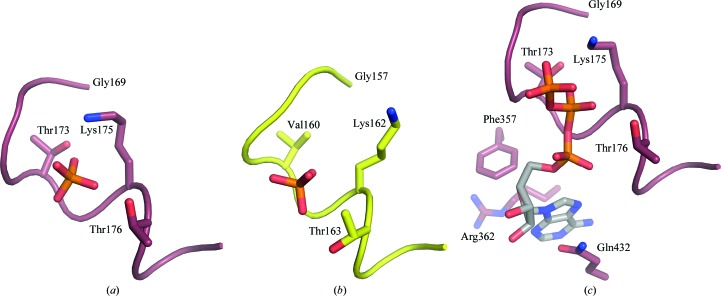
Association of a phosphate ion with the phosphate-binding or P-loops of the α-subunit from *P. denitrificans* and of the β_E_-subunit from bovine F_1_-ATPase inhibited with the ATP analogue AMP-PNP (adenylylimidodiphosphate; PDB entry 1h8h; Menz *et al.*, 2001 ▸). (*a*) The P-loops of the α-subunit from *P. denitrificans* (residues 169–176) shown in deep red and (*b*) the P-loops of the the bovine β-subunit (residues 157–163) shown in yellow. The bound phosphate ions are shown in orange and red. In (*c*), for reference, ATP is shown bound to the nucleotide-binding site of the α_E_-subunit of bovine F_1_-ATPase (PDB entry 1h8h).

Although there are no nucleotides associated with the *P. denitrificans* α_E_β_E_ catalytic dimer, electron density interpreted as a phosphate ion is associated with the phosphate-binding loop or P-loop region (residues 169–176) in the nucleotide-binding domain of the α-subunit. It is bound *via* interactions with residues Thr173, Gly174, Lys175 and Thr176 (Fig. 5 ▸). The P-loop is a feature of many NTPases, and is so named because it interacts with phosphate moieties of bound NTP or NDP molecules (Walker *et al.*, 1982 ▸). Neither phosphate nor sulfate was present in any of the buffers employed in the purification and crystallization processes, and it probably arises from hydrolysis of ATP in the purification and crystallization buffers.

Phosphate has not been observed bound in the vicinity of the P-loop regions of α-subunits in other structures of F_1_-ATPase. However, electron density in the β_E_-subunit adjacent to the P-loop has been interpreted as either a phosphate or a sulfate ion in the structures of bovine F_1_-ATPase in the ground state (Abrahams *et al.*, 1994 ▸; PDB entry 1bmf), in complexes inhibited with beryllium fluoride (Kagawa *et al.*, 2004 ▸; PDB entry 1w0j) or azide (Bowler *et al.*, 2006 ▸; PDB entry 2ck3) and in the complex of F_1_-ATPase and the peripheral stalk sub­complex (Rees *et al.*, 2009 ▸; PDB entry 2wss). However, the anion-binding site in the β_E_ P-loop is about 8 Å from where the γ-phosphate of the substrate ATP is bound in the catalytically active β_DP_-subunit and from where presumably phosphate is released following scission of the bond between the β- and γ-phosphates (Bason *et al.*, 2015 ▸; PDB entry 4yxw). Currently, there is no experimental evidence supporting the involvement of a phosphate ion bound in the vicinity of the β_E_ P-loop of F_1_-ATPase in the catalytic mechanism of the enzyme.

### 3.4. Significance of the structure   

The F-ATPase from *P. denitrificans* is an attractive target for further structural and functional study, especially because the mechanism of the regulation of its ATP hydrolase activity involving the ζ inhibitor protein is not understood. The intact enzyme has been crystallized and diffraction data have been collected (Morales-Ríos *et al.*, 2015 ▸). The current structure should be helpful in the interpretation of the structural data for the intact F-ATPase.

## Supplementary Material

PDB reference: the α/β dimer of the F-ATPase from *Paracoccus denitrificans*, 5cdf

## Figures and Tables

**Table 1:** Crystallographic data-collection and refinement statistics Values in parentheses are for the highest resolution bin.

Space group	*P*2_1_
Unit-cell parameters ()	*a* = 72.6, *b* = 102.9, *c* = 89.2
Resolution range ()	33.552.30 (2.372.30)
No. of unique reflections	48901
Multiplicity	2.9 (2.8)
Completeness (%)	91.4 (94.1)
*R*_merge_ †	0.137 (0.525)
*I*/(*I*)	5.5 (1.9)
*B* factor from Wilson plot ()^2^	25.7
*R* factor‡ (%)	22.5
Free *R* factor§ (%)	25.7
R.m.s.d., bond lengths ()	0.007
R.m.s.d., angles ()	1.06

† *R*_merge_ = 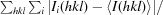 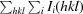, where *I*(*hkl*) is the mean weighted intensity after the rejection of outliers.

‡ *R* factor = 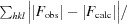 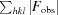, where *F*_obs_ and *F*_calc_ are the observed and calculated structure-factor amplitudes, respectively.

§ *R*_free_ = 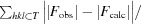 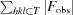, where *F*_obs_ and *F*_calc_ are the observed and the calculated structure-factor amplitudes, respectively, and *T* is the test set of data omitted from refinement.
